# Neurolymphomatosis as a manifestation of relapsed diffuse large B‐cell lymphoma

**DOI:** 10.1002/jha2.536

**Published:** 2022-10-17

**Authors:** Ikue Okamura‐Shiki, Koiku Asakura, Takashi Ikeda

**Affiliations:** ^1^ Division of Haematology and Stem Cell Transplantation Shizuoka Cancer Center Shizuoka Japan; ^2^ Division of Diagnostic Radiology Shizuoka Cancer Centre Shizuoka Japan

**Keywords:** diffuse large B‐cell lymphoma, Neurolymphomatosis, peripheral mononeuropathy

1

A 49‐year‐old woman presented with right drop foot and desensitization in the peroneal region. She had been diagnosed with stage IE primary cutaneous diffuse large B‐cell lymphoma (PCLBCL), leg type, 13 months ago. Five months ago, she had achieved complete remission after receiving six cycles of R‐CHOP chemotherapy (rituximab, cyclophosphamide, doxorubicin, vincristine, and prednisolone), as determined using 18‐F fluorodeoxyglucose positron emission tomography/computed tomography (^18^F‐FDG‐PET/CT). She consulted an orthopedic physician and was diagnosed with unidentified right peroneal neuropathy. Her symptom neither aggravation nor improved. However, 8 months after onset, she presented with cutaneous nodules on the right foot. Nodule biopsy led to a diagnosis of PCLBCL relapse. ^18^F‐FDG‐PET/CT imaging showed increased metabolism in the right inguinal lymph node, right sural nerve, right tibial nerve, right fibula nerve, and right sciatic nerve (Figure 1, left image and upper right image). Contrast‐enhanced magnetic resonance imaging (MRI) showed diffuse enlargement with an enhancement of the right sciatic nerve and right tibial nerve on T1‐weighted images (Figure 1, bottom right image). Owing to the risk of irreversible neurological deficits, we did not perform a nerve biopsy. Neurolymphomatosis with relapsed PCLBCL appeared highly probable. There was no lymphomatous infiltration of the meninges on cerebrospinal fluid (CSF) cytopathologic evaluation or central neuraxis on MRI. The patient was treated with conventional chemotherapy, CHASER (rituximab, cyclophosphamide, cytarabine, etoposide, and dexamethasone). After two cycles of CHASER, ^18^F‐FDG‐PET/CT showed no abnormal uptake; however, there was no clinical improvement with regard to deficits of right peroneal neuropathy because of the prolonged neural damage. Complete remission was achieved with conventional chemotherapy; therefore, the patient planned to receive myeloablative chemotherapy with autologous stem cell transplantation.

**FIGURE 1:**
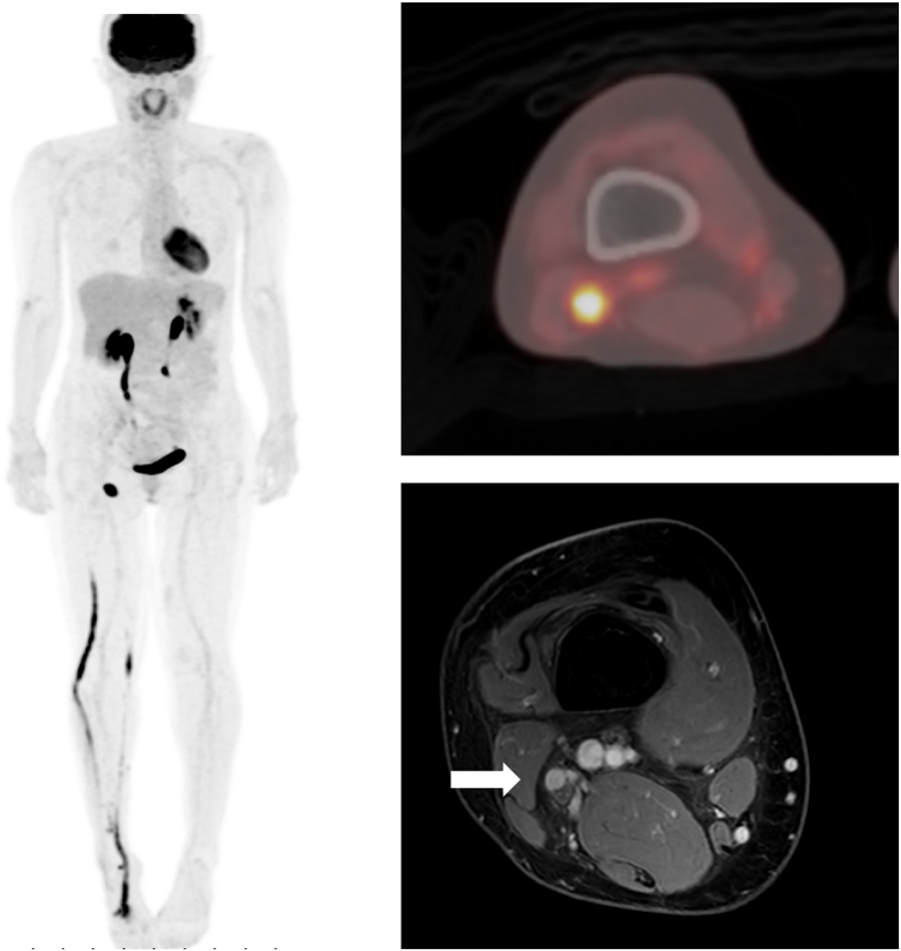
18‐F fluorodeoxyglucose positron emission tomography/computed tomography (^18^F‐FDG‐PET/CT) imaging showed increased metabolism in the right inguinal lymph node, right sural nerve, right tibial nerve, right fibula nerve, and right sciatic nerve (left image and upper right image). Contrast‐enhanced magnetic resonance imaging (MRI) showed diffuse enlargement with an enhancement of the right sciatic nerve and right tibial nerve on T1‐weighted images (bottom right image).

Neurolymphomatosis is defined as direct endoneurial invasion by lymphoma cells, such as that of the cranial nerves, peripheral nerve roots, plexus, or peripheral nerves [1]. It occurs in approximately 5% of patients with lymphomas, with a strong preponderance of diffuse large B‐cell lymphomas [1]. Four clinical patterns have been recognized. Most commonly, neurolymphomatosis presents as painful polyneuropathy or polyradiculopathy, followed by cranial neuropathy, painless polyneuropathy, and peripheral mononeuropathy [2]. Its diagnosis requires the integration of clinical information; imaging findings (MRI and ^18^F‐FDG‐PET/CT); and morphologic data obtained from neural tissue, nonneural tissue, and CSF. Especially in the absence of systemic lymphoma, the diagnosis is often delayed, as in our case. As the index manifestation of recurrent lymphoma, neurolymphomatosis should always be considered. Its successful treatment is contingent upon recognition of the condition and its exact neuroanatomic localization without delay.

## CONFLICT OF INTEREST

The authors declare no conflict of interest.

## FUNDING INFORMATION

The authors received no funding for this study.

## AUTHOR CONTRIBUTIONS

TI monitored the patient. KS performed radiological investigations. IS and TI wrote the report.

## ETHICS STATEMENT

Written consent to publish this case was obtained from the patient.

## Data Availability

Data sharing is not applicable to this report as no datasets were generated or analyzed during the current study.
